# Effective saturation pulse for the whole heart at 3 T

**DOI:** 10.1186/1532-429X-11-S1-P105

**Published:** 2009-01-28

**Authors:** KellyAnne McGorty, Daniel Kim

**Affiliations:** New York University Langone Medical Center, New York, NY USA

**Keywords:** Right Ventricle, Saturation Pulse, Residual Magnetization, Effective Saturation, Proton Density Weighted Image

## Introduction

Cardiac MRI at 3 T is a promising modality to increase the contrast-to-noise ratio (CNR) in first-pass cardiac perfusion imaging. This CNR boost can improve the accuracy of perfusion estimation from dynamic contrast-enhanced images. However, radio-frequency (RF) field (B_1_) variations and dielectric effects are comparatively higher at 3 T than at 1.5 T. These challenging factors make it difficult to perform accurate T_1_-weighting using a conventional rectangular saturation pulse. Previous studies have shown improved saturation of magnetization at 3 T using adiabatic B_1_-insensitive rotation (BIR-4) [1], rectangular RF pulse train [1], and specific absorption rate (SAR) constrained rectangular RF pulse train [2]. Unfortunately, these pulses did not achieve effective saturation of magnetization, which we shall define as residual magnetization after saturation < 5% of equilibrium magnetization (M_0_), within the whole heart, while remaining within clinically acceptable SAR limits. The purpose of this study was to develop an adiabatic-rectangular pulse train at 3 T that can achieve both of the aforementioned objectives.

## Methods

### Pulse sequence

**Figure 1:**
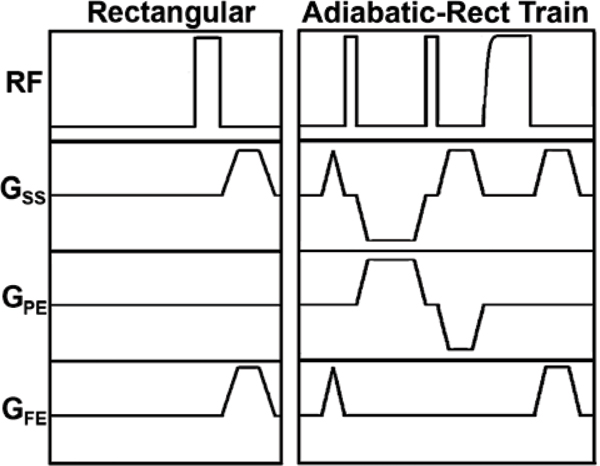
**Pulse sequence diagrams of rectangular pulse and adiabatic-rectangular pulse train**.

Figure 1 shows pulse sequence diagrams of a conventional rectangular pulse and an adiabatic-rectangular pulse train. This pulse train consists of a rectangular 130° pulse with 0.8 ms duration, a rectangular 85° pulse with 0.5 ms duration, and an adiabatic half-passage pulse with 1.5 ms duration. Crusher gradients were played between successive RF pulses to suppress stimulated echoes. The total pulse durations, excluding the final spoiler gradients, for the rectangular and adiabatic-rectangular pulse train were 1 ms and 8.8 ms, respectively. These two saturation pulses were implemented on a 3 T whole-body MR scanner (Tim-Trio; Siemens) equipped with an 12-channel phased array RF coil.

The residual longitudinal magnetization left behind by the saturation pulse can be measured by performing a saturation-no-recovery experiment using a TurboFLASH pulse sequence with centric k-space reordering, as previously described [1]. Normalization of the saturation image by the proton density weighted (PDW) image corrects for surface coil inhomogeneities and M_0_. Relevant imaging parameters include: field of view = 320–400 × 255–324 mm, acquisition matrix = 64 × 52, slice thickness = 8 mm, TE/TR = 1.1/2.3 ms, TD = 3 ms, image acquisition time = 78 ms, flip angle = 10°, parallel imaging acceleration factor = 1.5, and bandwidth = 1002 Hz/pixel. PDW image was acquired using 3° flip angle and without the saturation pulse. The RF and receiver scales were kept constant between the different acquisitions per subject.

### Cardiac imaging

Nine volunteers (5 males; 4 females) were imaged in 3 short-axis (apical, mid-ventricular, basal) views of the left ventricle (LV), 4-chamber view, and 2-chamber view of each ventricle.

### Image analysis

The LV and right ventricle (RV) were segmented manually. For each subject, the mean normalized signal within the whole heart was computed by averaging the normalized intensities from all five regions of interests (i.e. 3 short-axis and 2 long-axis views). Reported data represent the mean and standard deviation over subjects (n = 9). Student t-test was performed to compare the residual magnetization values between the two RF pulses.

## Results

**Figure 2:**
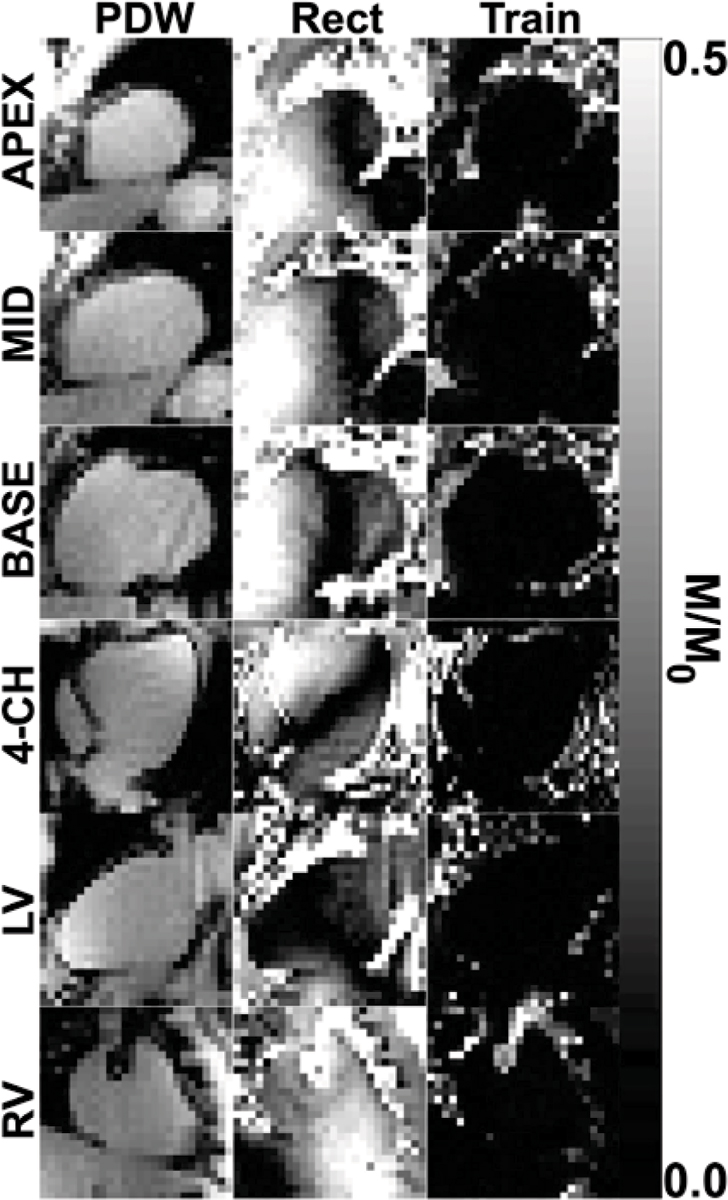
**Representative normalized saturation images in six different views of the heart: PDW** ***(left)*** **, rectangular pulse** ***(middle)*** **, and adiabatic-rectangular pulse** ***(right)***.

The energy of the adiabatic-rectangular pulse was 14.2 times larger than that of the rectangular pulse. Figure 2 shows representative saturation images that demonstrate their considerably different efficacies. Statistically, the adiabatic-rectangular pulse reduced the residual magnetization by 92% in the RV (0.03 ± 0.01 vs. 0.38 ± 0.08; p < 0.001, respectively) and 84% in the LV (0.02 ± 0.01 vs. 0.13 ± 0.04; p < 0.001, respectively) compared with the rectangular pulse.

## Discussion

This study demonstrates that the new adiabatic-rectangular pulse train can effectively saturate the magnetization within the whole heart at 3 T, while remaining within clinically acceptable SAR limits. This saturation pulse can be used to acquire multiple slices (>5) with our typical clinical first-pass perfusion protocols. Effective saturation of magnetization is likely to produce more accurate estimation of cardiac perfusion.
